# The naked mole rat genome: understanding aging through genome analysis

**DOI:** 10.18632/aging.100417

**Published:** 2011-12-26

**Authors:** Vadim N. Gladyshev, Guojie Zhang, Jun Wang

**Affiliations:** ^1^ Division of Genetics, Department of Medicine, Brigham and Woman's Hospital, Harvard Medical School, Boston, MA 02115, USA; ^2^ BGI-Shenzhen, Shenzhen, 518083, China

In the past 15 years, genomics has penetrated all areas of the life sciences [1], and this dramatic change in the way science is done is often viewed as genome revolution. However, application of genomics to study senescence and aging lagged behind. Until this year, no genomes have been sequenced explicitly to understand aging. In this regard, the recent completion of the genome of the naked mole rat [2] marks an important milestone, as this study was performed primarily to better understand the exceptional longevity of this rodent.

Naked mole rats live in the subterranean niche in southeast Africa and are the longest-lived rodents (maximum lifespan of 32 years). Being the size of a mouse, naked mole rats can be conveniently studied in the laboratory setting and compared with other rodents. Delayed aging is not the only exceptional trait in naked mole rats. These animals are also not known to develop cancer, do not maintain stable body temperature, can live at low oxygen and high carbon dioxide concentrations in the atmosphere, do not feel certain types of pain, and represent one of only two known eusocial mammals [3].

What have we learned from the initial analysis of the genome of this remarkable animal? First, the genome is characterized by the reduced level of polymorphism, consistent with low DNA mutation rate (although other explanations are also possible). Second, an unusual thermogenesis of naked mole rats, which may be linked to longevity through metabolic rate, is consistent with the altered sequence of a key thermoregulator, UCP1, which modified its sequences that mediate regulation by nucleotides and fatty acids. Another interesting finding is the unique sequence of a tumor suppressor p16, a protein whose mutations were linked to a variety of human diseases associated with aging. In addition, analyses of gene expression as a function of age distinguished naked mole rats from other mammals. Each of these as well as several other findings reported in the genome paper [2] provides interesting leads which can now be examined experimentally.

The availability of the genome of the naked mole rat should be viewed as the first step in the process of understanding of the delayed aging of this mammal, and also as a useful resource for studying the aging process in general. It is clear that much of its value lies in comparison with the genomes of other mammals, both with long and short lifespans, as well as in downstream functional genomic studies that assess genetic and epigenetic regulation networks. Comparative genomics of short-lived and long-lived organisms offers great opportunities to understand evolutionary forces and molecular mechanisms that regulate lifespan. Now that the cost of sequencing decreased dramatically, groups of related organisms with different lifespans can be sequenced and evaluated for differences in genome organization, genes, pathways and systems. It is also clear, however, that there are no easy ways to interpret these differences, so many genomes will need to be examined, and this activity should involve a broader research community. We are now entering an exciting time when aging can be understood through genome analyses.
